# Relationship between depression and dorsolateral prefronto-thalamic tract injury in patients with mild traumatic brain injury

**DOI:** 10.1038/s41598-020-76889-3

**Published:** 2020-11-12

**Authors:** Sung Ho Jang, Hyeok Gyu Kwon

**Affiliations:** 1 Department of Physical Medicine and Rehabilitation, College of Medicine, Yeungnam University, Daegu, Republic of Korea; 2 Department of Physical Therapy, College of Health Science, Eulji University, Sansungdaero 533, Sujung-gu, Sungnam-si, Gyeonggi 13135 Republic of Korea

**Keywords:** Neuroscience, Neurology

## Abstract

The prefrontal lobe has been considered to be closely related to depression. This study examined the relationship between depression and three prefronto-thalamic tract (PF-TT) regions (the dorsolateral prefronto-thalamic tract [DLPF-TT], ventrolateral prefronto-thalamic tract [VLPF-TT], and the orbitofronto-thalamic tract [OF-TT]) in patients with mild traumatic brain injury (TBI), using diffusion tensor tractography (DTT). Thirty-seven patients with depression following mild TBI were recruited based on Beck Depression Inventory-II (BDI-II) scores. Thirty-one normal control subjects were also recruited. The three regions of the PF-TTs were reconstructed using probabilistic tractography and DTT parameters for each of the three PF-TT regions were determined. The tract volume of the DLPF-TT and OF-TT in the patient group showed a significant decrease compared to that of the control group (*p* < 0.05). The BDI-II score of the patient group showed a moderate negative correlation with the tract volume value of the right (*r* =  − 0.33) and left (*r* =  − 0.41) DLPF-TT (*p* < 0.05). On the other hand, no significant correlations were detected between the BDI-II score of the patient group and the values of the other DTT parameters values for the three PF-TT regions (*p* > 0.05). Using DTT, depression was found to be closely related to a DLPF-TT injury in patients with mild TBI. We believe that evaluation of the DLPF-TT using DTT would be helpful when assessing patients with depression following mild TBI. These results can provide useful information regarding the proper application of neuromodulation in the management of depression.

## Introduction

Traumatic brain injury (TBI) may be classified into three types (mild, moderate, and severe) according to the level of injury severity^1^. Among these, mild TBI is observed in 75–90% of all TBI patients. Moreover, up to 44% of mild TBI patients were reported to suffer from depression^2,3^. Depression, a risk factor associated with poor recovery, leads to a range of problems, such as mood (anxiety, anger, and irritability), behavior (loss of interest and appetite and suicidal thinking), cognitive (memory and attention), physical (fatigue and headache), and sleep problems^1,3^. These problems can deteriorate a patient’s quality of life^4^. Therefore, it is important to investigate the pathogenic mechanism of depression. Previous studies related to the pathogenic mechanism of depression reported that depression is associated with multi-focal brain abnormalities including abnormalities in the cingulate cortex, amygdala, hippocampus, basal ganglia, thalamus, prefrontal cortex (PFC), and other brain regions^5,6^. In particular, the dorsolateral PFC (DLPFC), which manages the modulation and regulation of mood, is considered to play a key role in depression^5,7,8^. Many studies using various brain imaging methods, including positron emission tomography, functional magnetic resonance imaging (MRI), and voxel-based morphometry, have reported that injury of the DLPFC can lead to depression^7,9–16^. In addition, the DLPFC has been a primary treatment target region of repetitive transcranial magnetic stimulation used in the management of depression^17,18^.

Recent developments of diffusion tensor imaging (DTI), which measures the diffusion of water molecules, has allowed an investigation of the entire microstructural features of white matter in pre-defined regions of interest (ROIs)^19^. This also has the advantage of being able to detect even subtle changes of the white matter in the human brain^19^. DTI is suitable for use in an investigation of depression because depression is associated with various brain regions. Of particular note, diffusion tensor tractography (DTT), which is a derivative of DTI, provides for three-dimensional visualization and localization of neural tracts in the human brain^19,20^. Previously, three regions within the prefronto-thalamic tract (PF-TT), the dorsolateral prefronto-thalamic tract [DLPF-TT], the ventrolateral prefronto-thalamic tract [VLPF-TT] and the orbitofronto-thalamic tract [OF-TT] were reconstructed using DTT in normal subjects^21^. Many studies have used DTI to examine the changes in various brain regions in patients with depression, including the PFC, cingulate cortex, and amygdala^8,11,12^. Regarding TBI, many DTI studies of patients with post-traumatic stress disorder or depression have been reported^22–25^. Regarding the DTT, however, only one case study incorporated DTT in its investigation of depression in a patient with TBI^26^.

Mild TBI is not usually associated with lesions that can be detected by conventional brain magnetic resonance imaging (MRI)^27^. In contrast, since the introduction of DTI, many DTI-based studies could demonstrate axonal injury lesions that are related to the presence of various clinical features such as central pain, memory impairment, or motor weakness in mild TBI patients^28,29^. Regarding depression, one case study reported that a DLPF-TT injury was related to the presence of depression^26^. On the other hand, the regions within the PF-TT that may be related to depression in mild TBI have not demonstrated. This study hypothesized that, among three PF-TT regions, depression in patients with mild TBI would be associated with injury of the DLPF-TT.

In the current study, DTT was used to investigate the relationship between depression and tract injury in three PF-TT regions (DLPF-TT, VLPF-TT, and OF-TT) in patients with mild TBI.

## Methods

### Subjects

Fifty-eight patients with mild TBI visited the rehabilitation department of a university hospital (Table 1). Among the 58 patients (male: 20, female: 38, mean age: 47.3 ± 13.4 years, range: 13–68 years), thirty-seven (male: 12, female: 25, mean age: 45.7 ± 9.2 years, range: 23–58 years) were recruited according to the following inclusion criteria: (1) loss of consciousness for < 30 min, post-traumatic amnesia for ≤ 24 h, and an initial Glasgow Coma Scale score of 13–15; (2) no specific lesion observed on brain MRI (T1-weighted, T2-weighted, and fluid-attenuated inversion recovery images); (3) more than one month had elapsed after the onset of TBI; (4) age range of 20–59 years; (5) no history of previous head trauma, or neurological or psychiatric disease; (6) presence of depression on Beck Depression Inventory-II score (BDI-II, a full score of 63, a cutoff value of 13; a higher score means more severe depression)^30^. Values of BDI-II were obtained by self-report. In addition, 37 healthy control subjects (male: 12, female: 25, mean age: 45.6 ± 10.5 years, range: 24–59 years) with no previous history of neurological, physical, or psychiatric illness were recruited. There are no significant differences in sex, mean age, and whole brain volume between patient and control groups. All healthy subjects understood the purpose of the study and provided written, informed consent before participation. Because this study was conducted retrospectively, we could not obtain patient's consent and the study protocol without patient's consent was approved by the Institutional Review Board of a Yeungnam university hospital. (YUMC-2019-06-032). The study was carried out in accordance with relevant guidelines and regulations.

**Table 1:** Demographic and clinical data of the patient and control groups.

	Patient group	Control group
Sex (male:female)	12:25	12:25
Mean age, years	45.7 (9.2)	45.6 (10.5)
LOC, minutes	4.3 (7.5)	–
PTA, min	10.5 (17.5)	–
GCS score	14.9 (0.1)	–
Mean duration to DTI, months	10.9 (11.5)	–
Whole brain volume (voxels)	251,477.86 (31,264.91)	262,314.77 (29,165.38)
**BDI-II severity, n (%)**		
Minimal	3 (8.1)	–
Mild	6 (16.2)	
Moderate	9 (24.3)	
Severe	19 (51.4)	
BDI-II score	30.1 (12.6)	

Values represent mean (± standard deviation).

*LOC* loss of consciousness, *PTA* post-traumatic amnesia, *GCS* Glasgow Coma Scale, *DTI* diffusion tensor imaging, *BDI* Beck Depression Inventory.

### Diffusion tensor tractography

DTI data were acquired an average of 10.9 ± 11.5 months after the onset of head trauma using a six-channel head coil on a 1.5 T Philips Gyroscan Intera with 32 non-collinear diffusion sensitizing gradients (Philips, Ltd., Best, Netherlands). The imaging parameters were as follows: acquisition matrix = 96 × 96; reconstructed to matrix = 192 × 192; field of view = 240 × 240 mm^2^; repetition time = 10,398 ms; echo time = 72 ms; parallel imaging reduction factor (SENSE factor) = 2; echo-planar imaging factor = 59; b = 1000 s/mm^2^; and a slice thickness of 2.5 mm (acquired voxel size 1.25 × 1.25 × 2.5 mm^3^). The head motion effects and image distortions due to the eddy current were corrected by applying an affine multi-scale two-dimensional registration using Functional Magnetic Resonance Imaging of the Brain (FMRIB) Software Library (FSL; www.fmrib.ox.ac.uk/fsl)^31^. One analyzer (Kwon HG) performed fiber tracking using probabilistic tractography and applied the default tractography option (5000 streamline samples, 0.5 mm step lengths, curvature thresholds = 0.2) of the Oxford Centre for FMRIB Software Library with BedpostX method^20,31,32^. For reconstruction of the PF-TT regions a seed ROI was placed on the known anatomical location of the mediodorsal nucleus of the thalamus on the coronal image of the b0 map on each subject’s brain and each of the target ROIs as follows^21^: (1) DLPFC as Brodmann areas (BAs) 8, 9, and 46 on the coronal image of the b0 map; (2) ventrolateral PFC as BAs 44, 45, and 47 on the coronal image of the b0 map; and (3) orbitofrontal cortex as BAs 47/12, 10, 11, and 13 on the axial image of the b0 map. Values of fractional anisotropy (FA), mean diffusivity (MD), and tract volume (TV) were determined for each subject using MATLAB™ (Matlab R2007b, The Mathworks, Natick, MA, USA).

### Statistical analysis

Statistical analyses were performed using SPSS software (v. 25.0; SPSS, Chicago, IL, USA). The independent *t* statistics were used to evaluate the differences between the DTT parameters (FA, MD, and TV values) of the three types of PF-TT regions (DLPF-TT, VLPF-TT, and OF-TT) in the patient and control groups. The r-statistic (Pearson correlation) was used to assess the relationships between the BDI-II score and the FA, MD, and TV values. The correlation coefficient (*r*) indicates the relative strength (0.1–0.3: weak, 0.3–0.5: moderate, > 0.5: strong) and direction (+ ,  −) of a linear relationship between the two values. The significance level of the obtained p value was set at 0.05.

## Results

**Figure 1:**
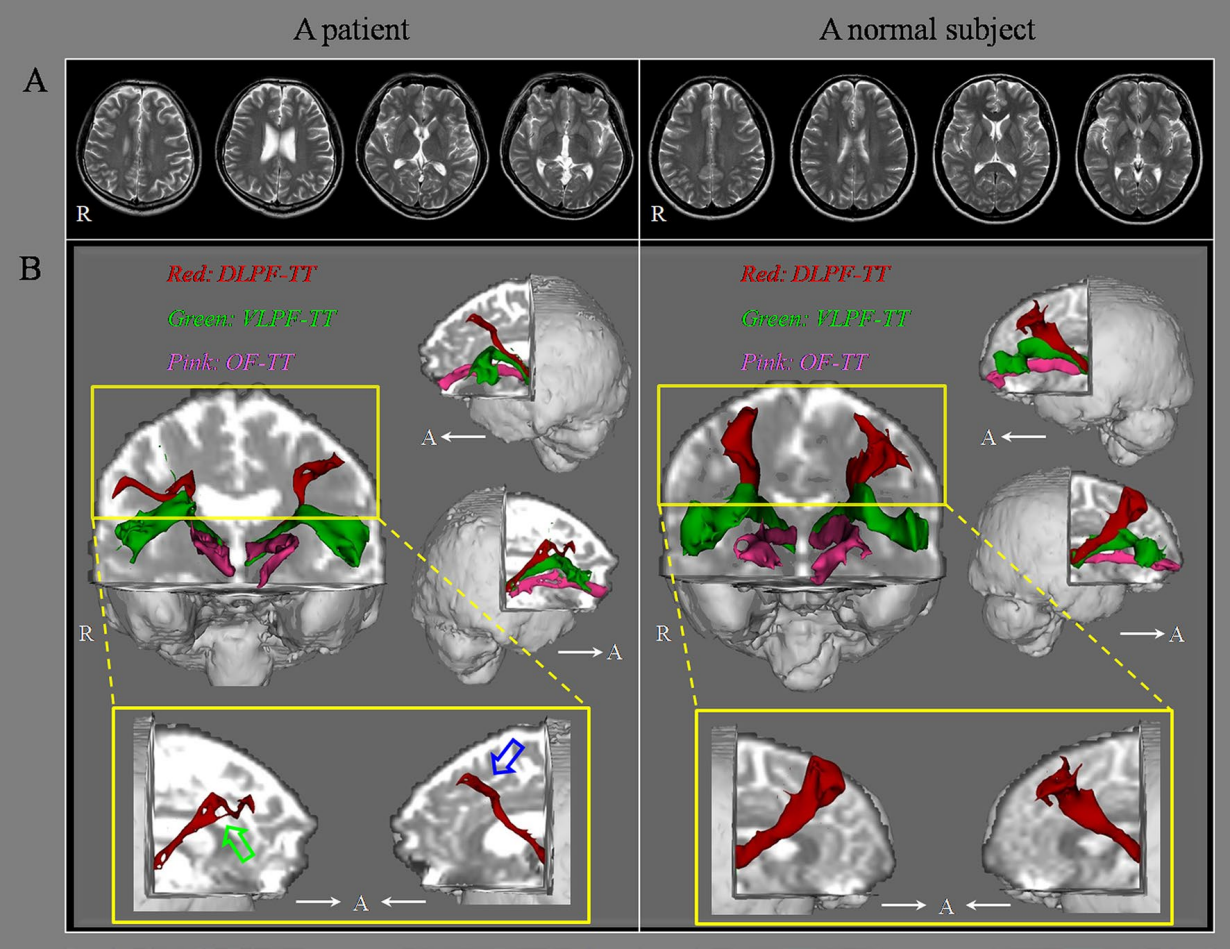
(**A**) T2-weighted brain magnetic resonance images showing no abnormalities in a representative patient (35-year-old male) and a normal subject (37-year-old male). (**B**) Results for the three prefronto-thalamic tract (PF-TT) regions on diffusion tensor tractography: DLPF-TT—dorsolateral prefronto-thalamic tract (red), VLPF-TT—ventrolateral prefronto-thalamic tract (green), and OF-TT—orbitofronto-thalamic tract (pink). The DLPF-TT in a patient shows partial tearing (green arrow) in the right hemisphere and narrowing (blue arrow) in the left hemisphere compared that that in a normal subject.

Table 2 presents a summary of the DTT parameters values for each of the three PF-TT regions as well as the correlations between the BDI-II score and DTT parameters of the three PF-TT regions (Table 2; Fig. 1). Regarding the DLPF-TT and OF-TT, the TV values of the patient group were significantly lower than those of the control group (*p* < 0.05). In contrast, there were no significant differences in the FA and MD values of the DLPF-TT and OF-TT between the patient and control groups (*p* > 0.05). In addition, there were no significant differences in the FA, MD, and TV values of the VLPF-TT between the patient and control groups (*p* > 0.05).

**Table 2:** Diffusion tensor tractography parameter values of the prefronto-thalamic tracts of the patient and control groups.

	Patient group			Control group		
	FA	MD	TV	FA	MD	TV
DLPF-TT	0.34 (0.03, 0.26)	0.81 (0.05, − 0.23)	449.35* (321.39, − 1.51)	0.34 (0.02)	0.81 (0.03)	1058.18 (179.03)
VLPF-TT	0.34 (0.03, 0.15)	0.83 (0.04, − 0.24)	1079.80 (384.96, − 0.37)	0.34 (0.02)	0.84 (0.05)	1082.08 (220.65)
OF-TT	0.36 (0.03, − 0.34)	0.80 (0.03, − 0.04)	570.20* (406.26, − 1.02)	0.36 (0.03)	0.80 (0.04)	994.26 (260.86)

Correlation between Beck Depression Inventory-II and DTT parameters						
	FA		MD		TV	
	Right	Left	Right	Left	Right	Left
DLPF-TT	0.248	0.228	− 0.235	− 0.254	− 0.328*	− 0.407*
VLPF-TT	0.255	0.175	− 0.101	− 0.076	− 0.213	− 0.034
OF-TT	0.143	0.206	− 0.030	− 0.259	− 0.249	− 0.005

Values represent mean (± standard deviation, effect size).

*FA* fractional anisotropy, *MD* mean diffusivity, *TV* tract volume, *DLPF-TT* dorsolateral prefronto-thalamic tract, *VLPF-TT* ventrolateral prefronto-thalamic tract, *OF-TT* orbitofronto-thalamic tract, *DTT* diffusion tensor tractography.

*Significant differences between patient and control groups, p < 0.05.

With regard to the correlation results, the BDI-II score of the patient group showed a moderate negative correlation with the TV values of the right (*r* =  − 0.33) and left (*r* =  − 0.41) DLPF-TTs (*p* < 0.05). On the other hand, no significant correlations were detected between the BDI-II score of the patient group and the other DTT parameter values of each of the three PF-TT regions (i.e., FA and MD of the DLPF-TT; FA, MD, and TV of the VLPF-TT and OF-TT) (*p* > 0.05).

## Discussion

This study examined the relationship between depression and three regions of the PF-TT in patients with mild TBI. The results can be summarized as follows. (1) The TV values of the DLPF-TT and OF-TT in the patient group were lower than those of the control group. (2) The BDI-II score showed a moderate negative correlation with TV value of the DLPF-TT. Among the commonly assessed DTT parameters, the FA, MD, and TV have been used wildly to demonstrate the status of various neural tracts. The FA value indicates the degree of directionality of water diffusion. Consequently, it reflects the fiber density, axonal diameter, and white matter myelination, whereas the MD value indicates the magnitude of water diffusion^33^. In contrast, TV is a measure of the number of voxels contained within a neural tract. As a result, it is an indication of the number of neural fibers within a neural tract^34^. Therefore, the relatively low TV values in these patients were indicative of tract injuries of the DLPF-TT and OF-TT. Regarding the BDI-II correlation results, the results suggest that the severity of depression is closely related to the severity of the injury to the DLPF-TT, particularly TV.

Several studies have reported the presence of DLPFC injury in patients with depression^7,9–16^. These studies showed that the blood flow and gray matter volume of the DLPFC in patients with depression were lower than those in the control subjects^7,9,10,13,14^. Regarding DTI analyses, a few studies reported decreases in the FA value in the DLPFC area, which was measured using an ROI-based method, of patients with depression compared to those of the control subjects^8,11,12^. Specifically, Taylor et al. (2004) and Yang et al. (2007) demonstrated that the superior frontal gyrus including the DLPFC exhibited a lower FA value in patients with late-life depression than the normal subjects^8,11^. In addition, Blood et al. (2010) reported that DLPFC white matter in patients with major depressive disorder had a lower FA value compared to that of normal subjects^12^. With regard to DTT, only one case study has been reported. In 2016, Jang et al. reported the presence of DLPF-TT injuries in both hemispheres of a patient with depression^26^.

To the best of our knowledge, this is the first original study to demonstrate a relationship between depression and DLPF-TT injuries in patients with mild TBI by using DTT. However, some limitations of this study should be considered. First, only patients with depression who visited the rehabilitation department of a university hospital were recruited. Therefore, it is possible that among the patients with mild TBI, we recruited patients with a different level of severity of clinical manifestations than that of other patient populations. Second, regarding the results of significance of only TV (non-significance of FA and MD), we could not provide the apparent evidence why non-significance of FA and MD were shown. Third, their answer for BDI-II could have been exaggerating because it was self-reported. In addition, the generalizability of the results was affected by the spectrum bias, which refers to the differential performance of a test in different settings^35^. Fourth, although DTT is a powerful anatomic imaging tool that can demonstrate gross fiber architecture, it can produce false positive and false negative results due to crossing fibers or the partial volume effect^36^. Therefore, further studies will be needed to overcome the above limitations.

In conclusion, by using DTT, we observed that depression was closely related to the presence of DLPF-TT injury in patients with mild TBI. We believe that DTT-based evaluation of the DLPF-TT would be helpful for patients with depression following mild TBI. In addition, although these results can provide information that may be useful for determining the proper application of neuromodulation, such as repetitive transcranial magnetic stimulation or transcranial direct current stimulation, in the management of depression, TBI-related depression is different than from major depressive disorders in terms of the seizure risk^37^.

## Data Availability

The dataset used and/or analyzed during current study are available from the corresponding author on reasonable request.
